# Supplementary material for: Enriched phenotypes in rare variant carriers suggest pathogenic mechanisms in rare disease patients
Source: BioData Min. 2025 Jan 17;18:6. doi: 10.1186/s13040-024-00418-5 (PMC11740427; doi:10.1186/s13040-024-00418-5)
Supplement: Supplementary file 1 — Supplementary Material 1. [file 13040_2024_418_MOESM1_ESM.pdf]

Supplemental Table 1: Seizure and Seizure-Related HPO Terms

**HPO**	**Name**
HP:0001250	Seizure
HP:0007359	Focal-onset seizure
HP:0033259	Non-motor seizure
HP:0032679	Focal non-motor seizure
HP:0020219	Motor seizure
HP:0011146	Dialeptic seizure
HP:0011153	Focal motor seizure
HP:0002384	Focal impaired awareness seizure
HP:0002349	Focal aware seizure
HP:0032680	Focal cognitive seizure
HP:0032716	Focal impaired awareness non-motor seizure
HP:0032682	Focal aware non-motor seizure
HP:0032807	Neonatal seizure
HP:0032808	Neonatal seizure with electrographic correlate
HP:0002133	Status epilepticus
HP:0032809	Neonatal electro-clinical seizure
HP:0011154	Focal autonomic seizure
HP:0032898	Focal automatism seizure
HP:0011157	Focal sensory seizure
HP:0032712	Focal impaired awareness motor seizure
HP:0020217	Focal aware motor seizure
HP:0025613	Focal emotional seizure

HP:0032813	Neonatal electro-clinical motor seizure
HP:0002197	Generalized-onset seizure
HP:0032756	Focal impaired awareness cognitive seizure
HP:0032681	Focal aware cognitive seizure
HP:0032677	Generalized-onset motor seizure
HP:0020207	Reflex seizure
HP:0020221	Clonic seizure
HP:0032755	Focal impaired awareness autonomic seizure
HP:0032909	Focal impaired awareness automatism seizure
HP:0032740	Focal aware autonomic seizure
HP:0032910	Focal aware automatism seizure
HP:0032794	Myoclonic seizure
HP:0032787	Focal impaired awareness sensory seizure
HP:0002266	Focal clonic seizure
HP:0032754	Focal aware sensory seizure
HP:0032892	Infection-related seizure
HP:0032746	Focal impaired awareness emotional seizure
HP:0032734	Focal aware emotional seizure
HP:0002069	Bilateral tonic-clonic seizure
HP:0032894	Seizure precipitated by febrile infection
HP:0002121	Generalized non-motor (absence) seizure
HP:0011097	Epileptic spasm
HP:0032792	Tonic seizure

HP:0032825	Neonatal electro-clinical sequential seizure
HP:0032815	Neonatal electro-clinical myoclonic seizure
HP:0012004	Focal cognitive seizure with deja vu/jamais vu
HP:0010819	Atonic seizure
HP:0020216	Visually-induced seizure
HP:0032821	Neonatal electro-clinical tonic seizure
HP:0032829	Neonatal electro-clinical motor seizure with automatism
HP:0032814	Neonatal electro-clinical clonic seizure
HP:0002123	Generalized myoclonic seizure
HP:0011166	Focal myoclonic seizure
HP:0002373	Febrile seizure (within the age range of 3 months to 6 years)
HP:0011145	Symptomatic seizures
HP:0033052	Psychogenic non-epileptic seizure
HP:0032812	Neonatal electro-clinical non-motor seizure
HP:0032903	Focal vocal automatism seizure
HP:0032904	Focal verbal automatism seizure
HP:0032907	Focal undressing automatism seizure
HP:0011167	Focal tonic seizure
HP:0032905	Focal sexual automatism seizure
HP:0011165	Focal sensory seizure with visual features
HP:0032759	Focal sensory seizure with vestibular features
HP:0011163	Focal sensory seizure with somatosensory features
HP:0011161	Focal sensory seizure with olfactory features

HP:0032760	Focal sensory seizure with hot-cold sensations
HP:0011160	Focal sensory seizure with gustatory features
HP:0032810	Focal sensory seizure with cephalic sensation
HP:0011158	Focal sensory seizure with auditory features
HP:0032902	Focal perseverative automatism seizure
HP:0032901	Focal pedal automatism seizure
HP:0032899	Focal orofacial automatism seizure
HP:0011175	Focal motor seizure with version
HP:0032721	Focal motor seizure with paresis/paralysis
HP:0032846	Focal motor seizure with negative myoclonus
HP:0032718	Focal motor seizure with dystonia
HP:0032720	Focal motor seizure with dysarthria/anarthria
HP:0032900	Focal manual automatism seizure
HP:0032725	Focal impaired awareness clonic seizure
HP:0011174	Focal hyperkinetic seizure
HP:0007332	Focal hemifacial clonic seizure
HP:0006813	Focal hemiclonic seizure
HP:0032906	Focal head nodding automatism seizure
HP:0032729	Focal emotional seizure with pleasure
HP:0032737	Focal emotional seizure with paranoia
HP:0010821	Focal emotional seizure with laughing
HP:0032739	Focal emotional seizure with fear/anxiety/panic
HP:0010820	Focal emotional seizure with crying

HP:0032736	Focal emotional seizure with anger
HP:0032727	Focal emotional seizure with agitation
HP:0032696	Focal cognitive seizure with receptive dysphasia/aphasia
HP:0032693	Focal cognitive seizure with neglect
HP:0032687	Focal cognitive seizure with memory impairment
HP:0032700	Focal cognitive seizure with left-right confusion
HP:0012008	Focal cognitive seizure with illusion
HP:0012007	Focal cognitive seizure with hallucination
HP:0032692	Focal cognitive seizure with forced thinking
HP:0032702	Focal cognitive seizure with expressive dysphasia/aphasia
HP:0032694	Focal cognitive seizure with dyslexia/alexia
HP:0032699	Focal cognitive seizure with dysgraphia/agraphia
HP:0032691	Focal cognitive seizure with dyscalculia/acalculia
HP:0032689	Focal cognitive seizure with dissociation
HP:0032698	Focal cognitive seizure with conduction dysphasia/aphasia
HP:0032685	Focal cognitive seizure with auditory agnosia
HP:0032701	Focal cognitive seizure with anomia
HP:0032715	Focal bilateral motor seizure
HP:0011173	Focal behavior arrest seizure
HP:0032711	Focal aware clonic seizure
HP:0032765	Focal autonomic seizure with urge to urinate/defecate
HP:0032763	Focal autonomic seizure with pupillary dilation/constriction
HP:0032767	Focal autonomic seizure with piloerection

HP:0032773	Focal autonomic seizure with palpitations/tachycardia/bradycardia/asystole
HP:0032762	Focal autonomic seizure with pallor/flushing
HP:0032771	Focal autonomic seizure with lacrimation
HP:0032766	Focal autonomic seizure with hypoventilation/hyperventilation/altered respiration
HP:0032764	Focal autonomic seizure with erection
HP:0011159	Focal autonomic seizure with epigastric sensation/nausea/vomiting/other gastrointestinal phenomena
HP:0020220	Focal atonic seizure
HP:0025190	Bilateral tonic-clonic seizure with generalized onset
HP:0011147	Typical absence seizure
HP:0032795	Generalized myoclonic-tonic-clonic seizure
HP:0032678	Eyelid myoclonia seizure
HP:0011172	Complex febrile seizure
HP:0007334	Bilateral tonic-clonic seizure with focal onset
HP:0020215	Thinking-induced seizure
HP:0020214	Startle-induced seizure
HP:0020213	Somatosensory-induced seizure
HP:0011171	Simple febrile seizure
HP:0033349	Seizure cluster
HP:0032665	Repeated focal motor seizures
HP:0500173	Reflex asystolic syncope
HP:0020212	Reading-induced seizure
HP:0033053	Pseudoseizure
HP:0020211	Proprioceptive-induced seizure

HP:0020210	Praxis-induced seizure
HP:0007207	Photosensitive tonic-clonic seizure
HP:0032855	Photosensitive myoclonic-tonic-clonic seizure
HP:0001327	Photosensitive myoclonic seizure
HP:0031951	Nocturnal seizures
HP:0032834	Neonatal seizure with unilateral automatism
HP:0032835	Neonatal seizure with bilateral symmetric automatism
HP:0032830	Neonatal seizure with bilateral asymmetric automatism
HP:0032816	Neonatal multifocal myoclonic seizure
HP:0032820	Neonatal multifocal clonic seizure
HP:0032824	Neonatal focal tonic seizure
HP:0032817	Neonatal focal myoclonic seizure
HP:0032818	Neonatal focal clonic seizure
HP:0032811	Neonatal electrographic only seizure
HP:0032823	Neonatal electro-clinical seizure with behavior arrest
HP:0032822	Neonatal electro-clinical autonomic seizure
HP:0032828	Neonatal bilateral symmetric tonic seizure
HP:0032836	Neonatal bilateral symmetric myoclonic seizure
HP:0032819	Neonatal bilateral clonic seizure
HP:0032831	Neonatal bilateral asymmetric tonic seizure
HP:0032832	Neonatal bilateral asymmetric myoclonic seizure
HP:0011150	Myoclonic absence seizure
HP:0032896	Music-induced seizure

HP:0031165	Multifocal seizures
HP:0032827	Multifocal neonatal sequential seizure
HP:0032786	Migrating focal seizure
HP:0100622	Maternal seizure
HP:0002173	Hypoglycemic seizures
HP:0002199	Hypocalcemic seizures
HP:0020209	Hot water-induced seizure
HP:0010818	Generalized tonic seizure
HP:0011170	Generalized myoclonic-atonic seizure
HP:0011169	Generalized clonic seizure
HP:0032887	Generalized atonic seizure
HP:0032893	Gastroenteritis-related afebrile seizure
HP:0032662	Focal-onset seizure evolving into bilateral convulsive status epilepticus
HP:0011168	Focal seizure with eyelid myoclonia
HP:0032826	Focal neonatal sequential seizure
HP:0032923	Focal impaired awareness vocal automatism seizure
HP:0032924	Focal impaired awareness verbal automatism seizure
HP:0032927	Focal impaired awareness undressing automatism seizure
HP:0032724	Focal impaired awareness tonic seizure
HP:0032925	Focal impaired awareness sexual automatism seizure
HP:0032806	Focal impaired awareness sensory seizure with visual features
HP:0032805	Focal impaired awareness sensory seizure with vestibular features
HP:0032890	Focal impaired awareness sensory seizure with somatosensory features

HP:0032804	Focal impaired awareness sensory seizure with olfactory features
HP:0032853	Focal impaired awareness sensory seizure with hot-cold sensations
HP:0032897	Focal impaired awareness sensory seizure with gustatory features
HP:0032878	Focal impaired awareness sensory seizure with cephalic sensation
HP:0032880	Focal impaired awareness sensory seizure with auditory features
HP:0032879	Focal impaired awareness seizure with dissociation at onset
HP:0032922	Focal impaired awareness perseverative automatism seizure
HP:0032921	Focal impaired awareness pedal automatism seizure
HP:0032918	Focal impaired awareness orofacial automatism seizure
HP:0032730	Focal impaired awareness myoclonic seizure
HP:0032713	Focal impaired awareness motor seizure with version
HP:0032859	Focal impaired awareness motor seizure with paresis/paralysis
HP:0032858	Focal impaired awareness motor seizure with negative myoclonus
HP:0032717	Focal impaired awareness motor seizure with dystonia
HP:0032719	Focal impaired awareness motor seizure with dysarthria/anarthria
HP:0032920	Focal impaired awareness manual automatism seizure
HP:0032726	Focal impaired awareness hyperkinetic seizure
HP:0032847	Focal impaired awareness hemifacial clonic seizure
HP:0032799	Focal impaired awareness hemiclonic seizure
HP:0032926	Focal impaired awareness head nodding automatism seizure
HP:0032747	Focal impaired awareness emotional seizure with pleasure
HP:0032749	Focal impaired awareness emotional seizure with paranoia
HP:0032750	Focal impaired awareness emotional seizure with laughing

HP:0032752	Focal impaired awareness emotional seizure with fear/anxiety/panic
HP:0032751	Focal impaired awareness emotional seizure with crying
HP:0032748	Focal impaired awareness emotional seizure with anger
HP:0032753	Focal impaired awareness emotional seizure with agitation
HP:0032793	Focal impaired awareness cognitive seizure with receptive dysphasia/aphasia
HP:0032798	Focal impaired awareness cognitive seizure with neglect
HP:0032801	Focal impaired awareness cognitive seizure with memory impairment
HP:0032796	Focal impaired awareness cognitive seizure with left-right confusion
HP:0032872	Focal impaired awareness cognitive seizure with illusion
HP:0032885	Focal impaired awareness cognitive seizure with hallucination
HP:0032888	Focal impaired awareness cognitive seizure with forced thinking
HP:0032886	Focal impaired awareness cognitive seizure with expressive dysphasia/aphasia
HP:0032870	Focal impaired awareness cognitive seizure with dyslexia/alexia
HP:0032803	Focal impaired awareness cognitive seizure with dysgraphia/agraphia
HP:0032802	Focal impaired awareness cognitive seizure with dyscalculia/acalculia
HP:0032882	Focal impaired awareness cognitive seizure with deja vu/jamais vu
HP:0032852	Focal impaired awareness cognitive seizure with conduction dysphasia/aphasia
HP:0032874	Focal impaired awareness cognitive seizure with auditory agnosia
HP:0032791	Focal impaired awareness cognitive seizure with anomia
HP:0032714	Focal impaired awareness bilateral motor seizure
HP:0032790	Focal impaired awareness behavior arrest seizure
HP:0032774	Focal impaired awareness autonomic seizure with urge to urinate/defecate
HP:0032779	Focal impaired awareness autonomic seizure with pupillary dilation/constriction

HP:0032772	Focal impaired awareness autonomic seizure with piloerection
HP:0032788	Focal impaired awareness autonomic seizure with palpitations/tachycardia/bradycardia/asystole
HP:0032777	Focal impaired awareness autonomic seizure with pallor/flushing
HP:0032782	Focal impaired awareness autonomic seizure with lacrimation
HP:0032775	Focal impaired awareness autonomic seizure with hypoventilation/hyperventilation/altered respiration
HP:0032780	Focal impaired awareness autonomic seizure with erection
HP:0032778	Focal impaired awareness autonomic seizure with epigastric sensation/nausea/vomiting/other gastrointestinal phenomena
HP:0032728	Focal impaired awareness atonic seizure
HP:0032915	Focal aware vocal automatism seizure
HP:0032916	Focal aware verbal automatism seizure
HP:0032908	Focal aware undressing automatism seizure
HP:0032722	Focal aware tonic seizure
HP:0032917	Focal aware sexual automatism seizure
HP:0032851	Focal aware sensory seizure with visual features
HP:0032800	Focal aware sensory seizure with vestibular features
HP:0032884	Focal aware sensory seizure with somatosensory features
HP:0032797	Focal aware sensory seizure with olfactory features
HP:0032877	Focal aware sensory seizure with hot-cold sensations
HP:0032889	Focal aware sensory seizure with gustatory features
HP:0032873	Focal aware sensory seizure with cephalic sensation
HP:0032864	Focal aware sensory seizure with auditory features
HP:0032914	Focal aware perseverative automatism seizure
HP:0032913	Focal aware pedal automatism seizure

HP:0032911	Focal aware orofacial automatism seizure
HP:0032758	Focal aware myoclonic seizure
HP:0032891	Focal aware motor seizure with version
HP:0032732	Focal aware motor seizure with paresis/paralysis
HP:0032857	Focal aware motor seizure with negative myoclonus
HP:0032723	Focal aware motor seizure with dystonia
HP:0032733	Focal aware motor seizure with dysarthria/anarthria
HP:0032912	Focal aware manual automatism seizure
HP:0032731	Focal aware hyperkinetic seizure
HP:0032854	Focal aware hemifacial clonic seizure
HP:0032757	Focal aware hemiclonic seizure
HP:0032919	Focal aware head nodding automatism seizure
HP:0032742	Focal aware emotional seizure with pleasure
HP:0032741	Focal aware emotional seizure with paranoia
HP:0032745	Focal aware emotional seizure with laughing
HP:0032738	Focal aware emotional seizure with fear/anxiety/panic
HP:0032743	Focal aware emotional seizure with crying
HP:0032735	Focal aware emotional seizure with anger
HP:0032744	Focal aware emotional seizure with agitation
HP:0032710	Focal aware cognitive seizure with receptive dysphasia/aphasia
HP:0032848	Focal aware cognitive seizure with neglect
HP:0032686	Focal aware cognitive seizure with memory impairment
HP:0032706	Focal aware cognitive seizure with left-right confusion

HP:0032704	Focal aware cognitive seizure with illusion
HP:0032871	Focal aware cognitive seizure with hallucination
HP:0032705	Focal aware cognitive seizure with forced thinking
HP:0032850	Focal aware cognitive seizure with expressive dysphasia/aphasia
HP:0032707	Focal aware cognitive seizure with dyslexia/alexia
HP:0032709	Focal aware cognitive seizure with dysgraphia/agraphia
HP:0032690	Focal aware cognitive seizure with dyscalculia/acalculia
HP:0032688	Focal aware cognitive seizure with dissociation
HP:0032883	Focal aware cognitive seizure with deja vu/jamais vu
HP:0032876	Focal aware cognitive seizure with conduction dysphasia/aphasia
HP:0032684	Focal aware cognitive seizure with auditory agnosia
HP:0032708	Focal aware cognitive seizure with anomia
HP:0032856	Focal aware bilateral motor seizure
HP:0032789	Focal aware behavior arrest seizure
HP:0032781	Focal aware autonomic seizure with urge to urinate/defecate
HP:0032768	Focal aware autonomic seizure with pupillary dilation/constriction
HP:0032783	Focal aware autonomic seizure with piloerection
HP:0032784	Focal aware autonomic seizure with palpitations/tachycardia/bradycardia/asystole
HP:0032761	Focal aware autonomic seizure with pallor/flushing
HP:0032776	Focal aware autonomic seizure with lacrimation
HP:0032769	Focal aware autonomic seizure with hypoventilation/hyperventilation/altered respiration
HP:0032770	Focal aware autonomic seizure with erection
HP:0032785	Focal aware autonomic seizure with epigastric sensation/nausea/vomiting/other gastrointestinal phenomena

HP:0020218	Focal aware atonic seizure
HP:0032895	Febrile seizure outside the age of 3 months to 6 years
HP:0020208	Eating-induced seizure
HP:0011152	Early onset absence seizures
HP:0006834	Developmental stagnation at onset of seizures
HP:0007193	Bilateral tonic-clonic seizure on awakening
HP:0032839	Bilateral symmetric neonatal sequential seizure
HP:0032837	Bilateral asymmetric neonatal sequential seizure
HP:0007270	Atypical absence seizure
HP:0011149	Absence seizure with eyelid myoclonia
HP:0012469	Infantile Spasms
HP:0200134	Epileptic encephalopathy
HP:0100284	EMG: myotonic discharges
HP:0032865	Myoclonic absence status epilepticus
HP:0032845	Focal aware epileptic spasms
HP:0032844	Focal impaired awareness epileptic spasm
HP:0032843	Focal-onset epileptic spasm
HP:0032842	Generalized-onset epileptic spasm
HP:0032841	Neonatal bilateral asymmetric epileptic spasm
HP:0032840	Neonatal bilateral symmetric epileptic spasm
HP:0032838	Neonatal unilateral epileptic spasm
HP:0032833	Neonatal epileptic spasm
HP:0032670	Tonic status epilepticus

HP:0032666	Hyperkinetic status epilepticus
HP:0032660	Convulsive status epilepticus
HP:0032549	Persistent asymmetrical tonic neck reflex
HP:0031954	Dystonic gait
HP:0031435	Monotonic speech
HP:0025097	Eyelid myoclonus
HP:0012074	Tonic pupil
HP:0007326	Progressive choreoathetosis
HP:0007166	Paroxysmal dyskinesia
HP:0007098	Paroxysmal choreoathetosis
HP:0007000	Morning myoclonic jerks
HP:0003730	EMG: myotonic runs
HP:0002487	Hyperkinetic movements
HP:0001336	Myoclonus
HP:0001332	Dystonia
HP:0001276	Hypertonia
HP:0001266	Choreoathetosis
HP:0000297	Facial hypotonia
