# Supplementary material for: Enriched phenotypes in rare variant carriers suggest pathogenic mechanisms in rare disease patients
Source: BioData Min. 2025 Jan 17;18:6. doi: 10.1186/s13040-024-00418-5 (PMC11740427; doi:10.1186/s13040-024-00418-5)
Supplement: Supplementary file 2 — Supplementary Material 2. [file 13040_2024_418_MOESM2_ESM.docx]

| Supplemental Table 2 | | | | | |
| --- | --- | --- | --- | --- | --- |
| **Gene** | **UDN Patient Phenotypes** | **Position** | **rsID** | **Frequency (GnomAD)** | **Consequence** |
| ATP7B | Dystonia  Gait disturbance  Drooling  Developmental regression  Behavioral abnormality  Dysarthria  Progressive muscle weakness  Hypermetropia | chr13:51968544 | rs138427376 | 0.003423 | Missense |
|  |  | chr13:51940233 | rs377042977 | 0.004258 | Intron |
| CACNA2D2 | Cognitive disability  Seizures  Autism spectrum disorder | chr3:50364696 | rs150284749 | 0.001270 | Missense |
|  |  | chr3:50365459 | rs146587089 | 0.001091 | Missense |
|  |  | chr3:50488800 | rs558982816 | 0.000121 | Intron |
| COL27A1 | Smooth philtrum  Wide nasal bridge  Down slanted palpebral fissures  Dental malocclusion  Flat forehead  Lambdoidal craniosynostosis  Metopic synostosis  Low-set ears  Microtia  Absent speech  Generalized hypotonia  Poor coordination  Focal impaired awareness seizure  Intellectual disability  Communicating hydrocephalus  Chiari type I malformation  Scoliosis  Pes planus  Limited elbow movement  Bilateral decreased elbow rotation  Accessory carpal bones  Congenital knee dislocation  Congenital bilateral hip dislocation  Hypermetropia  Oligohydramnios | chr9:114169384 | rs144786117 | 0.000307 | Missense |
|  |  | chr9:114284346 | rs145837394 | 0.00184 | Intron |
| DENND2C | Increased CSF lactate  Color vision defect  Muscle weakness  Secondary amenorrhea  Mental deterioration  Memory impairment  Seizure  Hyperreflexia in upper limbs  Focal-onset seizure  Abnormal cerebral cortex morphology  Cerebral cortical atrophy | chr1:114623516 | rs199773157 | 0.000214 | Missense |
|  |  | chr1:114601566 | rs753435776 | 0.000014 | Missense |
| MPO | Seizures, generalized tonic-clonic, triggered by sleep deprivation  Developmental regression  Intellectual disability  Autism  Absent speech  Impaired social interactions  Focal emotional seizure with laughing  Abnormal autonomic nervous system physiology  Broad-based gait  Headache  Sleep disturbance  Recurrent infections  Recurrent candida infections  Iron deficiency anemia  Episodic vomiting  Chronic constipation  Gastroparesis  Aganglionic megacolon  Gastroesophageal reflux  Hiatus hernia  Gastrointestinal dysmotility  Hyperammonemia  Hiatus hernia  Gastrointestinal dysmotility  Neurogenic bladder  Urinary retention  Pruritus  Dermatographic urticaria  Obesity | chr17:58272835 | rs119468010 | 0.001733 | Missense |
|  |  | chr17:58270865 | rs35897051 | 0.004717 | Splice Acceptor |
| PIGQ | Seizure  Irritability  Dysphagia  EEG abnormality  Sleep disturbance  Abnormal head movements  Involuntary movements  Profound global developmental delay  Tongue thrusting  Cerebellar vermis hypoplasia  Inguinal hernia  Abdominal distention  Gastrostomy tube feeding in infancy  Feeding difficulties  Abdominal distention  Vesicoureteral reflux  Hydronephrosis  Microphallus(resolved)  Intestinal malrotation  Gastrointestinal dysmotility  Volvulus  Duodenal diverticula  Hand clenching  Axial hypotonia  Appendicular hypotonia | chr16:576255 | rs200661329 | 0.000057 | Splice Donor |
|  |  | chr16:574123 | rs149108761 | 0.00000 | Missense |
| P2RX7 | Narrow mouth  Thin upper lip vermilion  Triangular face  Anxiety  Bilateral tonic-clonic seizure  Delayed skeletal maturation  Clinodactyly of the 5th finger  Short stature  Diffuse white matter abnormalities  Moderate expressive language delay  Moderate receptive language delay  Short palpebral fissure  Cognitive impairment | chr12:121156133 | rs28360445 | 0.001390 | Missense |
| SQSTM1 | Microcephaly  Decreased lacrimation  Motor stereotypy  Inappropriate laughter  Global developmental delay  Encephalopathy  Dystonia  Constipation  Sleep disturbance  Decreased sweating | chr5:179807631 | rs376407400 | 0.001459 | Intron |
|  |  | chr5:179836445 | rs104893941 | 0.001291 | Missense |
